# Genomic variability of *Mycoplasma hyopneumoniae* within pig lung lobes

**DOI:** 10.1186/s40813-021-00195-1

**Published:** 2021-01-28

**Authors:** Matteo Tonni, M. Beatrice Boniotti, Sara Gasparrini, Flavia Guarneri, Nicoletta Formenti, Maria Pieters, Paolo Pasquali, Giovanni L. Alborali

**Affiliations:** 1 Istituto Zooprofilattico Sperimentale della Lombardia e dell’Emilia Romagna, Via Bianchi, 9, 25124 Brescia, Italy; 2 Department of Veterinary Population Medicine, and Veterinary Diagnostic Laboratory, University of Minnesota, 1365 Gortner Ave, St. Paul, MN 55108 USA; 3 Department of Food Safety, Nutrition and Veterinary Public Health, Istituto Superiore di Sanità viale, Regina Elena 299, 00161 Rome, Italy

**Keywords:** Swine, MLVA, VNTR type, *Mycoplasma hyopneumoniae*, Lung, Lobe, Variability

## Abstract

**Background:**

Genotypic variability in *M. hyopneumoniae* has been reported within and among herds. However, information regarding VNTR types within single lung lobes is lacking. The objective of his study was to analyse *M. hyopneumoniae* infections and their association with VNTR types and lung lesions at the lobe level. Lungs from 300 pigs from 10 farms experiencing an enzootic pneumonia outbreak were collected and scored. *M. hyopneumoniae* was detected by real-time PCR and genotyped by MLVA assay in all samples.

**Results:**

The results showed genotypic variability within single pigs and among lung lobes. At the lobe level, infection with one VNTR type (SN infection) was dominant. Lobes with lesion scores > 0 were associated with positive results for real-time PCR. At the lobe level, no relationship was observed between infections with more than one genotype (MX infections) and the proportion of *Mycoplasma*-like lesions. Lesion-free lobes presented a higher proportion of MX infections than lobes scored > 0. *M. hyopneumoniae* was detected more frequently in the right lobe of the lung (*p* < 0.05), with a similar distribution within lobes for SN and MX infections. The anatomic conformation of swine lungs led to a higher prevalence of infections in the right lobe. However, this study showed that this condition did not affect the distribution of infections with multiple VNTR types. Nevertheless, careful consideration of sample selection should be practised for *M. hyopneumoniae* genotype analyses, including lung lobes with no visible lesions.

**Conclusion:**

The results did not show a significant association between the number of detected genotypes and the severity of the lesions at the lung lobe level, but revealed the unexpected detection of *M. hyopneumoniae* genotypes in lesion-free lobes. These results imply that a representative sampling of all lobes may lead to an accurate identification of the VNTR-type distribution. Further studies including factors that can affect pathogenetic evolution of this bacterium could shed light on the complexity of the relationship between genotypes and the lung lesions magnitude.

**Supplementary Information:**

The online version contains supplementary material available at 10.1186/s40813-021-00195-1.

## Background

*Mycoplasma hyopneumoniae* (*M. hyopneumoniae*) is the bacterial aetiological agent of enzootic pneumonia, a respiratory disease that causes significant economic losses in the pig industry [16]. Infections with *M. hyopneumoniae* are highly prevalent, chronic and detected in most countries [7]. Moreover, *M. hyopneumoniae* is one of the main pathogens of the porcine respiratory disease complex (PRDC). Pneumonic lesions caused by *M. hyopneumoniae* are predominantly well delineated in the cranioventral areas of the lung, involving the apical, middle, and accessory lobes, and in severe cases, lesions can extend and involve the caudal lobes [20].

Although it has a small genome, *Mycoplasma* harbours several variable numbers of tandem repeat (VNTR) regions [13]. VNTRs include numerous regions in adhesion protein genes, which can be investigated through genetic characterization [18]. Multiple locus variable number tandem repeat analysis (MLVA) is the preferred technique to investigate VNTRs and has been applied in various studies to characterize *M. hyopneumoniae* [2, 3, 12, 22] based on its high discriminatory power. The molecular variability of *M. hyopneumoniae* among farms has been the subject of several studies, and a wide range of heterogeneity has been reported, depending on management, production system [5, 15] and geographical distribution [3, 11, 19].

Currently, *M. hyopneumoniae* genomic classification and the corresponding terminology follow the definition of VNTR types [1]. Preliminary studies of the association between the severity of *Mycoplasma*-like lung lesions and the number of different genotypes revealed no association [2] or a positive association [12]. Nevertheless, the literature is based on analyses at the herd level or at the lung level, and the distribution of *M. hyopneumoniae* within each lung lobe and how it can affect the magnitude and localization of lesions have not been considered. Therefore, the aim of this study was to analyse the presence of different *M. hyopneumoniae* VNTR types and the associations between single- or multiple-genotype infections and lung lesion observations at the lung lobe level.

## Results

The genotype analysis performed on samples collected from 300 pigs on ten farms (A-J) showed 29 different VNTR types (Table 1).

**Table 1:** Summary of pigs, lungs with *Mycoplasma*-like lesions and sampled lung lobes per farm. Genotyping of *Mycoplasma hyopneumoniae* on farms A-G showed the VNTR type detected in lobes with *Mycoplasma*-like lesions, while the data for farms H-J included all lobes sampled (including lesion-free lobes)

Farm ID	A	B	C	D	E	F	G	H	I	J
Pigs	30	30	30	30	30	30	30	30	30	30
Lungs with score > 0	19	25	15	10	22	14	17	21	29	20
Sampled lobes	47	85	41	14	58	64	118	116	186	117
VNTR type										
2								108	136	
12	20				53					
13	1				2					
17		35								
18		3								
27		29								
28				8						
29	1			2	2					
38			1							
46						12				
47						39				
48							58			
51				5						
52				1						
53				2						
54				2						
55				2						
56					1					
57					23					
65										44
66										50
67										37
69										11
70										1
72										23
75			1							
81			4							
85			8							
86			22							

### Genotypes of mycoplasma hyopneumoniae in lobes with scores > 0 (farms A-G)

Two hundred ten fattening pigs were selected from farms with evidence of a respiratory disease outbreak. The number of lungs with a lesion score > 0 was 122, while the total number of sampled lung lobes (with M*ycoplasma-*like lesions) was 427. Two hundred sixty lung lobes were positive for *M. hyopneumoniae* by real-time PCR, and the strains were completely genotyped and categorized as SN (79.2%) or MX (20.8%) infection at the lobe level. Seven samples that were nontypable by MLVA analysis and 31 lobes that lost integrity during the slaughtering procedure were excluded from the study and not replaced.

**Fig. 1:**
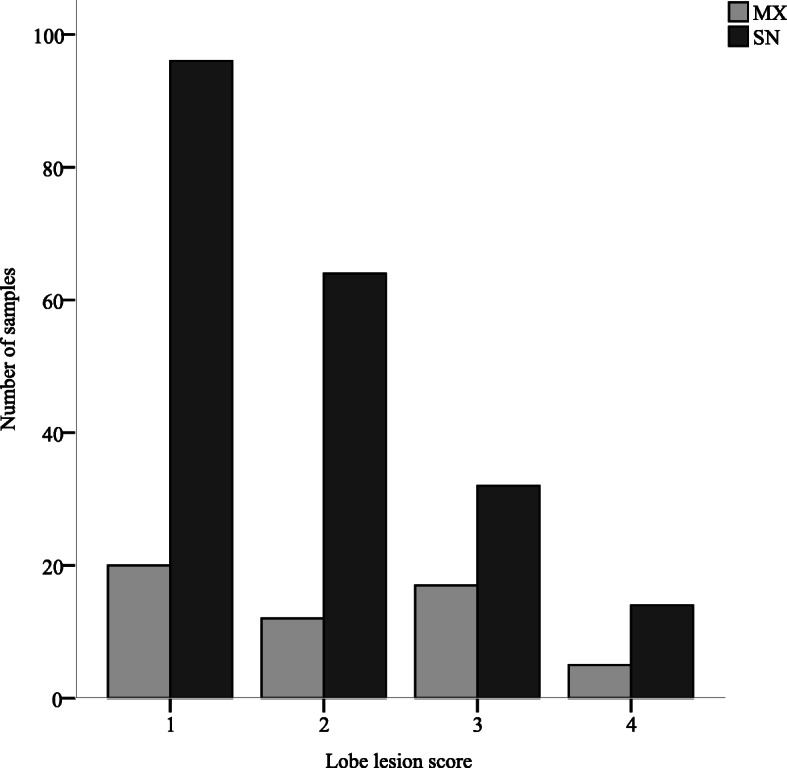
Distribution of lesion scores in lobes with single- or mixed-genotype infections (farms A-G). SN: Single-genotype infections. MX: Multiple-genotype infections. Lung lesion score 1–4 [6]

The lesion scores for lung lobes in which samples were genotyped are reported in Fig. 1. The majority of lobes scored 1 (44.6%) or 2 (29.3%), while the remaining lobes scored 3 (18.8%) and 4 (7.3%). Figure 1 also shows the genotype distribution between SN and MX. No significant association was detected between the magnitude of lesions and MX infections.

### Genotypes of mycoplasma hyopneumoniae in lobes with scores of 0–4 (farms H-J)

Seventy-one lungs (497 lobes), excluding in-farm mortality and lungs damaged during the slaughtering procedure, were collected at slaughterhouse and processed for analysis. *M. hyopneumoniae* was identified in 70/71 examined lungs. At the lobe level, the presence of M*ycoplasma*-like lesions was associated with positive real-time PCR results (p < 0.05), as reported in Table 2.

**Table 2:** Association between *Mycoplasma*-like lesions and detection of *Mycoplasma hyopneumoniae* by PCR at the lobe level (farms H-J)

	M*ycoplasma*-like lesions	
	Presence	Absence
PCR Positive	213	121
PCR Negative	39	46

**Fig. 2:**
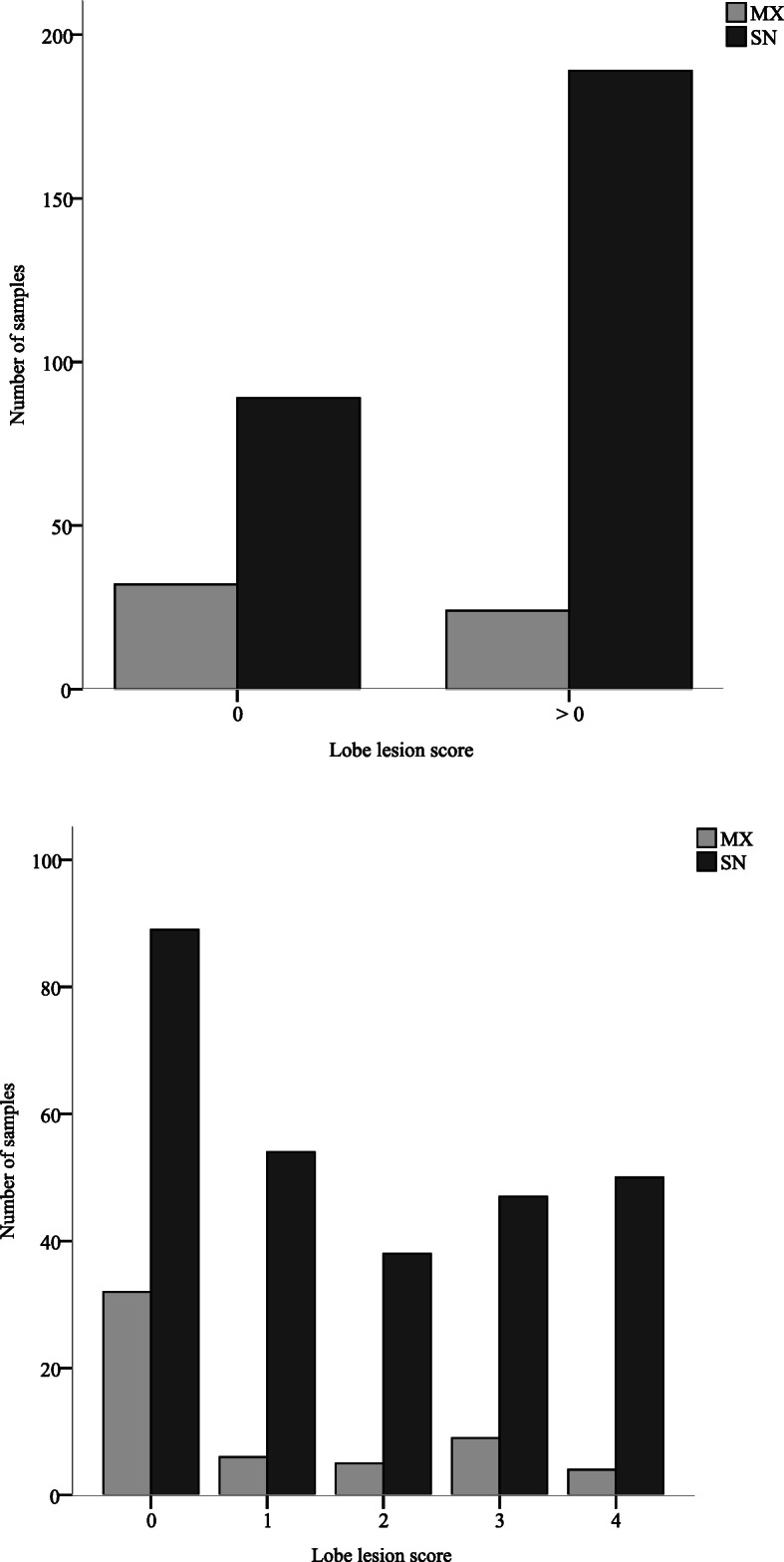
**a** Distribution of single- or mixed-genotype infections in lobes free of lesions (score = 0) and lobes with lesions (score > 0). Data from farms H-J. Lung lesion score = 0 or score > 0 [6]. **b** Distribution of lesion scores in lobes with single- or mixed-genotype infections (farms H-J). SN: Single-genotype infections. MX: Multiple-genotype infections. Lung lesion score 0–4 [6]

Real-time PCR and MLVA typing were performed on all lung lobes from all farms. Three hundred thirty-four samples were positive by real-time PCR, with 278 SN infections (83.2%) and 56 MX infections (16.8%), while the remaining 85 lobes were negative for the detection of *M. hyopneumoniae*. A significantly higher proportion of MX infection was recorded in lobes free of lesions (score = 0) than in lobes with lesions (score > 0; *p* < 0.001; Fig. 2a).

The lung lobe score assessment revealed 167 lobes free of lesions (39.9%), 73 lobes that scored 1 (17.4%), 52 lobes that scored 2 (12.4%), 67 lobes that scored 3 (16%) and 60 lobes that scored 4 (14.3%). The distribution of single- or mixed-genotype infections according to score (Fig. 2b) showed that MX infections accounted for 26.4% of infections in lobes with a score of 0 but 1.8, 5, 6, and 7.4% of infections in lobes with scores of 1, 2, 3 and 4, respectively. A significant relationship between MX genotype infections and lung lesion score was detected (*p* = 0.011). A higher probability of detecting MX genotype infections was observed in lobes free of lesions (score = 0) than in lobes with scores of 1 (*p* = 0.005) or 4 (*p* = 0.012).

**Fig. 3:**
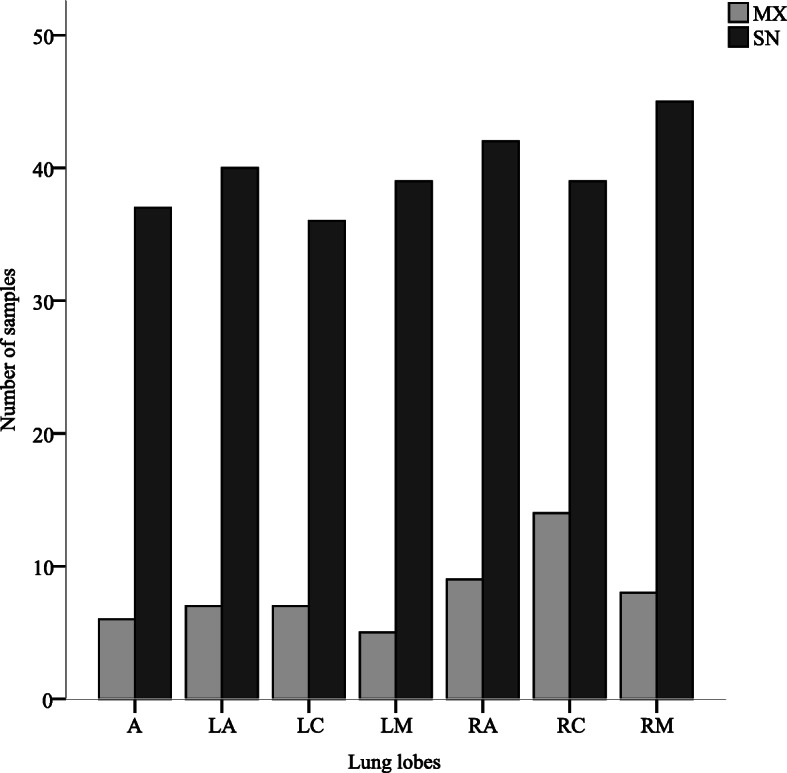
Distribution of single- and multiple-genotype infections of *Mycoplasma hyopneumoniae* within lung lobes (farms H-J). Single-genotype infections (SN). Multiple-genotype infections (MX). RA: Right apical lobe. LA: Left apical lobe. RM: Right middle lobe. LM: Left middle lobe. RC: Right caudal lobe. LC: Left caudal lobe. A: Accessory lobe

The collected dataset allowed us to investigate the topographical distribution of *M. hyopneumoniae* within lobes, for which the results are reported in Table 3. *M. hyopneumoniae* was detected more frequently on the right side of the lung (*p* < 0.05) in the RM, RC, and RA lobes. The distribution of SN or MX genotype infections within lung lobes was homogeneous, as shown in Fig. 3. No association was detected between the genotype of *M. hyopneumoniae* infections and individual lung lobes.

**Table 3:** Distribution of *Mycoplasma hyopneumoniae* real-time PCR results according to lung lobe (farms H-J)

Lung lobe	PCR Positive	PCR Negative
RA	51	11
LA	47	15
RM	53	6
LM	44	14
RC	53	9
LC	43	15
A	43	15

*RA* Right apical lobe, *LA* Left apical lobe, *RM* Right middle lobe, *LM* Left middle lobe, *RC* Right caudal lobe, *LC* Left caudal lobe, *A* Accessory lobe

## Discussion

The present study using VNTR typing based on four loci showed a high genomic variability of *M. hyopneumoniae* among the investigated population. The terminology and classification of the MLVA assay followed recently proposed criteria [1] specific for *M. hyopneumoniae*. The genomic variability observed in this study is consistent with what has been observed in previous studies, either in specific geographic regions [3] or at the farm level [11]. Genotypic variability of *M. hyopneumoniae* within a single pig has been reported [12]. Here, more detailed information is provided. Performing genotyping at the lobe level allowed an assessment of the genotypic variability within individual lung lobes, as well as the exact correspondence between visible lesions and the VNTR types that caused them. This approach provided evidence of what may occur within each scored lobe, which is different from the whole-lung view. In addition, our results suggest that each lung lobe is an independent unit; thus, in the *post-mortem* diagnostics a sampling method that involves all lung lobes is recommended. Furthermore, representative sampling should include lobes both with and without lesions. Therefore, when the entire lung is available, a pool of all lobes is preferred over single-lobe sampling.

Although high genotypic variability was observed among farms, few VNTR types were detected within a single herd, as previously reported by others [2, 10, 14, 22], suggesting a certain clustering distribution. The aforementioned studies suggested that the VNTR types belonging to each cluster exhibit minute differences in VNTR length, likely due to a difference of one tandem repeat sequence [1], and these differences have unclear biological significance. The clinical implications of clustering are not fully known, and future studies are needed to improve our understanding.

The relationship between genotypic variability and lung lesions remains controversial, considering either the entire organ or single lobes. In this study, no relationship was observed between the number of VNTR types and *Mycoplasma*-like lesions at the lobe level. This is in accordance with reports by Charlebois and colleagues at the lung level [2]. Conversely, other studies have shown that lungs with multiple infections seem to be associated with more severe lung lesions [12, 21, 22]. It can be speculated that different VNTR types could have specific virulence patterns. Hence, it may be reasonable to consider individual VNTR types and their associations in field infections.

It is known that VNTR types tend to be conserved at the farm level [2, 11], showing only minor variations [15]. However, studies regarding the pathogenic pathways associated with multiple infections within a single pig are lacking. *M. hyopneumoniae* is known to persist in the respiratory tract of pigs for long periods of up to 7 months [17]. Vranckx et al. [23] reported that *M. hyopneumoniae* persisted in the same animals and that infection with an *M. hyopneumoniae* VNTR type did not exclude secondary infection with another VNTR type 12 weeks later.

Although *M. hyopneumoniae* has been detected in scalding water, Marois et al. [9] showed that lungs from SPF pigs remained PCR negative after processing. Our research focused on lung lobe-related factors that had not been reported in the literature; therefore, we need to consider scalding water contamination as a possibility and as a limiting factor.

A high degree of association between positive outcomes for real-time PCR and *Mycoplasma*-like lesions was observed in this investigation. However, the proportion of lesion-free lung lobes positive for *M. hyopneumoniae* in real-time PCR suggests that all lobes should be considered for genomic characterization. Furthermore, an unexpectedly high proportion of MX infection was detected in lobes that scored zero. Further studies may be needed to clarify this phenomenon.

Considering each lobe individually, the majority of infections were single infections. Since different VNTR types can be identified at the lung level [12], our results may indicate that infections with *M. hyopneumoniae* within the lungs can be the result of a combination of VNTR types individually hosted in each lung lobe. This is likely due to the anatomic conformation of the pig lungs, which have deep fissures that separate the lobes from each other and lead to minimal collateral ventilation [20].

Although the topographical distribution of *M. hyopneumoniae* showed a slightly higher detection rate on the right side of the lungs [4], this association only approached the significance level. A potential explanation for this finding could be the anatomic framework of swine airways. The RA lobe receives airflow from the tracheal bronchus, located cranially to the main bronchi, which is vulnerable to early colonization by airborne pathogens [20]. Anatomic assumptions suggest considering each lobe as an independent anatomic unit, while our results showed marginal differences in lobe localization among VNTR types. Although the distribution of genotypes within the lung lobes appears homogeneous, the detection of these also in lesion-free lobes suggest that to obtain a full picture of all VNTR types, sampling of all lobes is required, regardless of the presence of *Mycoplasma*-like lesions.

Our study initially followed the IZSLER standard laboratory protocol and processed only lobes with scores > 0. This limitation was overcome for the results obtained on farms H-J, which extended sampling to all lobes.

The time when enzootic pneumonia outbreaks occur can influence the lesions observed at the slaughterhouse [7], and the Italian pig industry uses a long finishing phase. It is reasonable to suppose that there is a long time from *M. hyopneumoniae* outbreaks to slaughter or additional possible secondary outbreaks, with the aforementioned implications. Therefore, the peculiarity of the Italian production system does not allow a direct comparison with other European and non-European countries.

It is reasonable to assume that regardless of the time of infection, the genotypes detected at market age are influenced by different factors, such as the presence of other infections, that occurred between the outbreak and the time of slaughter. Therefore, multiple infections may be due to coinfection with more than one VNTR type during an enzootic pneumonia outbreak or due to sequential infections. Since the data collected in this study are not sufficient to reveal an accurate clinical history for each farm, the results must be interpreted taking this criticality into consideration.

It is important to note that clinical signs and the severity of lesions may depend on several variables that were not considered here, such as concomitant infections, farm management and environmental conditions [7]; conversely, a deeper knowledge of clinical farm history allows a better understanding of the dataset. As previously mentioned, the existence of different time frames from outbreak to slaughter affect the results [7], such as the excluded and untypable samples. All of these elements need to be considered limiting factors for this study.

For the reasons listed above and regardless of the *M. hyopneumoniae* genotyping method, unravelling the complex relation between genotypic variability at the lung or lobe level and lung lesions is a pressing need for proper disease control in the field. Further studies could shed light on the complexity of the abovementioned relationship, including investigation of the roles of other pathogens and the consequences of different farm management practices. Only complete information on the circulating *M. hyopneumoniae* genotypes can support a deep analysis of the effectiveness of control methods and approaches to enhance their effects.

## Conclusions

In conclusion, this study showed that VNTR types were highly variable among farms and within lungs, with the possibility of detecting single or multiple types. Moreover, a clear relation between the number of detected genotypes and the severity of the lesions at the lung lobe level has not been detected, but the presence of isolates in lesion-free lobes has been reported. These results imply that representative sampling of all lobes may lead to a more accurate view of the VNTR-type distribution. Further studies including factors that can affect pathogenetic evolution could shed light on the complexity of the abovementioned relationship.

## Methods

### Study design

The study was performed between June 2016 and June 2018, according to farm participation availability. Seven fattening farms (A-G) from a high-pig density area in Italy that reported clinical signs (mild, dry, nonproductive cough, respiratory distress, fever and slow growth) suggestive of an acute *M. hyopneumoniae* outbreak by the attending veterinarian were randomly selected from the regional epidemiological registry. The criterion for a farm to be included in the study was the detection of *M. hyopneumoniae* genetic material on at least seven out of ten tracheobronchial swabs per farm by PCR test, performed in the IZSLER laboratory or the histopathological evaluation of lung tissue samples collected during the outbreak. Samples were also tested for porcine reproductive and respiratory syndrome virus (PRRS), porcine circovirus type 2 (PCV2), swine influenza virus (SIV), *Actinobacillus pleuropneumoniae* (*A. pleuropneumoniae*), *Pasteurella multocida* (*P. multocida*), Streptococci and *Glässerella parasuis,* which showed negative results.

Based on the initial results, the same operating protocol was extended to consider all lobes, regardless of whether lesions were observed, for three farms (H-J), with the addition of a negative control. The selection of farms and pigs followed the same experimental design, but all the lung lobes were sampled, including lobes free of lesions, which allowed us to study the distribution of *M. hyopneumoniae* VNTR types in all lung lobes.

All pigs were vaccinated for PCV2, Aujeszky’s disease and *M. hyopneumoniae*; in particular, six farms (A, B, C, D, E and I) followed a one-shot vaccination protocol for *M. hyopneumoniae* with a single vaccination at 20–28 days, and four farms (F, G, H, J) followed a two-shot vaccination protocol, with vaccinations before and after weaning. Although treatment with tetracyclines or macrolides was administered due to the outbreak to all pigs (including those selected for research) no routine medication had been adopted at the farms. All farms belonged to a multisite production system with standard housing conditions characterized by a confined barn environment with natural ventilation.

During the course of the outbreak, groups of 30 fattening pigs per farm were randomly selected, regardless of clinical status, ensuring the bias-free selection of subjects. Then, lungs from the selected pigs were collected after slaughter. The age of the pigs at slaughter was approximately nine months for all farms. The standard procedure for the detection of *M. hyopneumoniae* infection included assessment of the lung score and PCR testing of all lobes with *Mycoplasma-*like lesions.

All samples from the ten farms (A-J) included in the study positive for *M. hyopneumoniae* by PCR were after genotyped by MLVA assay.

### Sample collection and lung scoring

Lungs were collected at slaughterhouses and transferred under refrigerated conditions (5 ± 3 °C) to the IZSLER laboratory, where the lungs were evaluated immediately, with the purpose of identifying lobes with lesions suggestive of porcine enzootic pneumonia. The presence of greyish to purplish consolidated pneumonic areas, primarily in the cranioventral parts of the lung, was considered suggestive of *M. hyopneumoniae* infection [7]. Lung lesions were scored following the method described by Madec and Kobisch [6] based on the magnitude (in percentage) of the affected lung lobe surface: 0% = no lesion, < 25% = score 1; 25–49% = score 2; 50–74% = score 3 and > 75% = score 4. A portion of the transition region between apparently healthy and affected areas of each injured lobe (farms A-J) and a portion of each apparently healthy lobe (farms H-J) were dissected. One gram of the sampled tissue was homogenized 1:10 in phosphate-buffered saline (PBS) and was maintained at −20 °C until analysis. Samples were sorted according to the labelled lobe, as follows: right apical (RA), right caudal (RC), right middle (RM), accessory (A), left apical (LA), left caudal (LC) and left middle (LM).

### DNA extraction, real-time PCR, and MLVA

Lung homogenates were centrifuged at 453 g for 10 min at 4 °C. Supernatants were used to obtain purified genomic DNA employing the DNeasy Blood Tissue Kit (Qiagen, Milan, Italy) according to the manufacturer’s protocol. Detection of *M. hyopneumoniae* was performed using a real-time PCR assay following routine IZSLER laboratory methods based on the protocol proposed by Marois et al. [8]. Briefly, the PCR mixture contained 1X Go Taq Probe Mastermix (Promega, Milan, Italy), 0.5 μM each primer, 0.2 μM p102 probe, 0.7x IPC Mastermix, 0.3X IPC DNA, and 5 μl of DNA and was brought to 4.6 μl with nuclease-free water. Amplification of DNA was carried out using a C1000 thermal cycler (BioRad®, Milan, Italy) using the following conditions: initial denaturation at 95 °C for 2 min and 40 cycles of denaturation at 95 °C for 3 s and annealing-extension at 60 °C for 20 s. The detection limit was set to 37 cycle threshold (Ct) values.

Samples positive by real-time PCR were MLVA genotyped through four conventional PCRs, one for each VNTR locus (Locus 1, Locus 2, P97-RR1 and P97-RR2), as described by Charlebois et al. [2]. The MLVA PCR mixture contained 5 μM each primer, 5 mM dNTPs, 0.5 U/μL Taq (Resnova, Rome, Italy), 5X Buffer solution, 12.7 μl of nuclease-free water and 5 μl of DNA. Amplification consisted of the following stages: 95 °C for 3 min, 40 cycles of 30 s at 95 °C, 30 s at 55 °C, and 1 min at 72 °C, and one final cycle of 72 °C for 1 min. MLVA typing was performed with an Applied Biosystems Thermocycler (Thermo Fisher Scientific, Milan, Italy). PCR products were analysed by capillary electrophoresis (QIAxcel, Qiagen). Samples with unclear results were further evaluated using a 2.0% high-resolution agarose gel, which was run at 100 V for 2 h and visualized under UV light. The size of the PCR products was calculated, and the approximate number of tandem repeats present was determined according to the allele-calling table (Additional file 1). An identification number was assigned to each VNTR profile type following the chronological order of identification at the IZSLER laboratory.

### Statistical and data analysis

Genotyping results at the lung and lobe levels were categorized as single infection (SN) or mixed infection (MX) based on the presence of one or more different VNTR types, respectively. Samples that were positive in real-time PCR for *M. hyopneumoniae* but had missing or unclear results for repeat sequences in MLVA analysis were excluded from the analysis. PCR-negative samples were identified as negative (NEG).

To investigate factors that may influence the distribution of single- or mixed-genotype infections, binomial generalized linear models (GLMs) were defined using SN/MX genotype infection as the response variable and farms, lung lobes, and lung lesion scores as explanatory variables.

The analyses were performed using SPSS Statistic 20.0 software. Values were significant when *p* < 0.05.

## Supplementary Information

**Additional file 1: Table 1.** Allele calling table. Each VNTR type is identified as a combination of different VNTR locus. The number of repeats of each locus is determinate by the number of base pair (bp) for that locus.

## Data Availability

The datasets used and/or analysed during the current study are available from the corresponding author upon reasonable request. The dataset supporting the conclusions of this article is included within the article.

## Abbreviations

PRDC: Porcine Respiratory Disease Complex
VNTRs: Variable Number of Tandem Repeats
MLVA: Multiple Locus Variable Number Tandem Repeat Analysis
SN: Single Infection
MX: Mixed Infection
RA: Right Apical
RC: Right Caudal
RM: Right Middle
A: Accessory
LA: Left Apical
LC: Left Caudal
LM: Left Middle
GLM: Generalized Linear Models
IZSLER: Istituto Zooprofilattico Sperimentale della Lombardia e dell’Emilia Romagna

## References

[1] Betlach AM, Maes D, Garza-Moreno L, Tamiozzo P, Sibila M, Haesebrouck F (2019). Mycoplasma hyopneumoniae variability: current trends and proposed terminology for genomic classification. Transbound Emerg Dis..

[2] Charlebois A, Marois-Créhan C, Hélie P, Gagnon CA, Gottschalk M, Archambault M (2014). Genetic diversity of mycoplasma hyopneumoniae isolates of abattoir pigs. Vet Microbiol.

[3] Dos Santos LF, Sreevatsan S, Torremorell M, Moreira MA, Sibila M, Pieters M (2015). Genotype distribution of mycoplasma hyopneumoniae in swine herds from different geographical regions. Vet Microbiol.

[4] Garcia-Morante B, Segalés JL, Fraile L, Pérez de Rozas A, Maiti H, Coll T, Sibila M (2016). Assessment of Mycoplasma hyopneumoniae-induced Pneumonia using Different Lung Lesion Scoring Systems: a Comparative Review. J Comp Pathol.

[5] Garza-Moreno L, Segalés J, Aragón V, Correa-Fiz F, Pieters M, Carmona M (2019). Characterization of mycoplasma hyopneumoniae strains in vaccinated and non-vaccinated pigs from Spanish slaughterhouses. Vet Microbiol.

[6] Madec K (1982). Bilan lesionnel des poumons de porcs charcutiers à ‘abattoir. Journees rech porcine en France.

[7] Maes D, Sibila M, Kuhnert P, Segalés J, Haesebrouck F, Pieters M (2018). Update on mycoplasma hyopneumoniae infections in pigs: knowledge gaps for improved disease control. Transbound Emerg Dis.

[8] Marois C, Dory D, Fablet C, Madec F, Kobisch M (2010). Development of a quantitative real-time TaqMan PCR assay for determination of the minimal dose of mycoplasma hyopneumoniae strain 116 required to induce pneumonia in SPF pigs. J Appl Microbiol.

[9] Marois C, Cariolet R, Morvan H (2008). H, Kobisch M, 2008, transmission of pathogenic respiratory bacteria to specific pathogen free pigs at slaughter. Vet Microbiol.

[10] Mayor D, Jores J, Korczak BM, Kuhnert P (2008). Multilocus sequence typing (MLST) of mycoplasma hyopneumoniae: a diverse pathogen with limited clonality. Vet Microbiol.

[11] Mayor D, Zeeh F, Frey J, Kuhnert P (2007). Diversity of mycoplasma hyopneumoniae in pig farms revealed by direct molecular typing of clinical material. Vet Res.

[12] Michiels A, Vranckx K, Piepers S, Del Pozo SR, Arsenakis I, Boyen F (2017). Impact of diversity of mycoplasma hyopneumoniae strains on lung lesions in slaughter pigs. Vet Res.

[13] Minion FC, Lefkowitz EJ, Madsen ML, Cleary BJ, Swartzell SM, Mahairas GG (2004). The genome sequence of mycoplasma hyopneumoniae strain 232, the agent of swine mycoplasmosis. J Bacteriol.

[14] Nathues H, Grosse Beilage E, Kreienbrock L, Rosengarten R, Spergser J (2011). RAPD and VNTR analyses demonstrate genotypic heterogeneity of mycoplasma hyopneumoniae isolates from pigs housed in a region with high pig density. Vet Microbiol.

[15] Pantoja LG, Pettit K, Dos Santos LF, Tubbs R, Pieters M (2016). Mycoplasma hyopneumoniae genetic variability within a swine operation. J Vet Diagn Investig.

[16] Pieters M, Maes D, Blackwell Publishing Group (2019). Mycoplasmosis. Diseases of Swine.

[17] Pieters M, Pijoan C, Fano E, Dee S (2009). An assessment of the duration of mycoplasma hyopneumoniae infection in an experimentally infected population of pigs. Vet Microbiol.

[18] Stakenborg T, Vicca J, Maes D, Peeters J, de Kruif A, Haesebrouck F (2006). Comparison of molecular techniques for the typing of mycoplasma hyopneumoniae isolates. J Microbiol Methods.

[19] Takeuti KL, de Barcellos DESN, de Andrade CP, de Almeida LL, Pieters M (2017). Infection dynamics and genetic variability of mycoplasma hyopneumoniae in self-replacement gilts. Vet Microbiol.

[20] Thacker EL, Minion C. Mycoplasmosis. In: Diseases of Swine. 10th ed. West Sussex: Wiley-Blackwell Ames; 2012. p. 779–97.

[21] Villarreal I, Maes D, Vranckx K, Calus D, Pasmans F, Haesebrouck F (2011). Effect of vaccination of pigs against experimental infection with high and low virulence mycoplasma hyopneumoniae strains. Vaccine..

[22] Vranckx K, Maes D, Calus D, Villarreal I, Pasmans F, Haesebrouck F (2011). Multiple-locus variable-number tandem-repeat analysis is a suitable tool for differentiation of mycoplasma hyopneumoniae strains without cultivation. J Clin Microbiol.

[23] Vranckx K, Maes D, Sacristán RP, Pasmans F, Haesebrouck F (2012). A longitudinal study of the diversity and dynamics of mycoplasma hyopneumoniae infections in pig herds. Vet Microbiol.

